# The prevalence and contextual correlates of non-communicable diseases among inter-provincial migrants and non-migrants in South Africa

**DOI:** 10.1186/s12889-021-11044-9

**Published:** 2021-05-27

**Authors:** Chukwuedozie K. Ajaero, Nicole De Wet-Billings, Chiemezie Atama, Prince Agwu, Eberechukwu J. Eze

**Affiliations:** 1 Department of Geography, University of Nigeria Nsukka, Nsukka, Nigeria; 2 Demography and Population Studies Programme, Schools of Public Health and Social Sciences, University of the Witwatersrand, Johannesburg, South Africa; 3 Department of Sociology/Anthropology, University of Nigeria Nsukka, Nsukka, Nigeria; 4 Department of Social Work, University of Nigeria Nsukka, Nsukka, Nigeria

**Keywords:** Correlates, Inter-provincial migration, Non-communicable diseases, Prevalence, South Africa

## Abstract

**Background:**

The socioeconomic conditions of different environments manifest in varying experiences of illnesses. Even as migrants do transit across these different environments for various reasons, including settlement, they are bound to have peculiar experiences of diseases, which could be traced to lifestyle, gender, adaptation, and reactions to specific social, economic, psychological and climatic conditions. Paying attention to such unique scenarios, our study examines the prevalence and contextual correlates of non-communicable diseases among inter-provincial migrants and non-migrants in South Africa.

**Methods:**

Data was from the National Income Dynamics Study (NIDS), waves 5 of 2017, which comprised of 28,055 respondents aged 15–64 years made up of 22,849 inter-provincial non-migrants and 5206 inter-provincial migrants. A composite dependent/outcome variable of non-communicable diseases (NCDs) was generated for the study and data analysis involved descriptive statistics, chi Square analysis and multilevel logistic regression analysis.

**Results:**

More migrants (19.81%) than non-migrants (16.69%) reported prevalence of NCDs. With the exception of household size for migrants and smoking for non-migrants, the prevalence of NCDs showed significant differences in all the community, behavioral, and individual variables. The factors in the full model, which significantly increased odds of NCDs among the migrants and the non-migrants, were older populations, the non-Blacks, and those with higher education levels. On the one hand, being married, having a household with 4–6 persons, and being residents of urban areas significantly increased odds of NCDs among the migrant population. While on the other, living in coastal provinces, being a female, and belonging to the category of those who earn more than 10,000 Rands were significantly associated with increased odds of NCDs among the non-migrants.

**Conclusions:**

These findings, therefore, among other things underscore the need for increased education and awareness campaigns, especially among the older populations on the preventive and mitigative strategies for NCDs. In addition, changes in lifestyles with regard to smoking and physical exercises should be more emphasized in specific contextual situations for the migrant and non-migrant populations, as highlighted by the results of this study.

## Background

Non-communicable diseases (NCDs) such as stroke**,** depression, cancer, diabetes, amongst others, contribute in no small measure to mortality and morbidity worldwide [1]. They comprise much of the world’s disease burden [2, 3]. However, there is ample evidence to show that NCDs are experienced differently across geographical, ethnic, and racial lines. For instance, 80% of mortality from NCDs occur in low- and middle-income countries (LMICs) [4]. Whilst the burden of NCDs in sub-Saharan African regions is higher than the global average and is now almost equivalent to the total burden associated with Communicable, Maternal, Neonatal, and Nutritional [CMNN] diseases [5]. Studies show that migrant and non-migrant experiences of diseases could differ, given prevailing social, cultural, political, and economic conditions [6, 7]. South Africa is the most significant destination point for migrants in Africa. It is reported by the International Organisation for Migration (IOM) that the country has over 4 million international migrants [8]. The implication of the much inflow of immigrants into South Africa, as in other parts of the world, could manifest in disease experiences, hence the need to investigate such implications regarding NCDs.

The four most common NCDs are cardiovascular disease (CVDs), including heart attack and stroke; cancers; chronic respiratory disease, including chronic obstructive pulmonary disease and asthma; and diabetes [1]. They are caused by a combination of modifiable and non-modifiable risk factors, including genetic, metabolic, behavioral and environmental factors [1]. It has been noted that the global epidemic of NCDs constitutes a public health emergency in slow motion [9]. Hence, in September 2011, at a United Nations high-level meeting on NCDs, heads of state and government formally recognized these diseases as a major threat to economies and societies and placed them high on the development agenda (2014). Subsequently, the World Health Organization (WHO) initiated a plan of action aimed at globally reducing mortality from cardiovascular disease, cancer, diabetes and chronic respiratory diseases by 25% before 2025 [10].

Literature show that South Africa grapple with “quadruple” burden of disease which are high level of HIV/AIDS, infectious diseases such as tuberculosis, high level of mortality and morbidity due to injuries and high levels of non-communicable disease (NCDs) [11]. Specifically, non-communicable diseases accounted for 43% of total deaths in all ages and sexes in South Africa in 2012 while the probability of dying between the ages 30–70 years due to NCD was 27% [10]. Evidence also shows that the burden of NCDs has increased over the past 15 years, resulting in an estimated 37% of all-cause mortality and 16% of disability adjusted life years [12].

The increased prevalence of NCDs in South Africa have been attributed to four lifestyle risk factors of poor diet, physical inactivity, tobacco use and inappropriate use of alcohol [11, 13]. In examining the differences in non-communicable disease risk factors in middle-income countries [14]. observed that alcohol consumption, patterns of smoking, and lack of physical activities are risk factors for NCDs [15]. equally reported the prevalence and variations in tobacco and alcohol use among migrants and non-migrant youths in South Africa. Migration has also been identified as a risk factor in the NCD epidemic, people migrate to urban or rural areas in search of greener pastures and this leads to changes in their lifestyle which may make them vulnerable to NCDS [13]. Changes in lifestyle behaviours accompanying migration are exemplified primarily by shifts in physical activity and dietary patterns which promote the development of obesity, diabetes, hypertension and cardiovascular diseases [16]. It has also been observed that nutritional patterns among migrants particularly in urban centres change rapidly with a shift to diets higher in fat, sugar and salt which have implications for NCDs [16]. In addition, understanding how urbanization and rural-urban migration influence risk-factors for noncommunicable disease (NCD) is crucial for developing effective preventative strategies [14].

Literature on the mental health of rural–urban migrants in developing countries show that after their initial physical health advantage, the migrants compared to the native populations become more vulnerable to various sources of stress, which lowers their mental health status [17–19]. In Bangladesh, [20] reported that international male migrants had comparable or lower injury and mortality risks than non-migrants. They also showed higher levels of self-rated health and physical strength but had substantially higher risks of overweight/obesity, hypertension, and depression. In Mexico, [21] found that internal migrants reported more anxiety, chronic fatigue, and pain. In addition, studies have shown that economically disadvantaged and socially isolated temporary migrants usually have more health and health-related behavioral problems compared to the native populations [22–26]. Gkiouleka et al. [27] study on the prevalence of depressive symptoms among migrant and non-migrant communities in 21 European countries showed that while the migrants had higher levels of depressive symptoms in seven of these countries, the migrants had lower levels of depressive symptoms in Greece and United Kingdom, compared to the non-migrant populations. According to [28] migrant adolescents showed greater health resilience than non-migrants. Tzogiou et al. [29] study showed that non-migrants are more likely to visit a doctor compared to first-generation and culturally different immigrants in Switzerland.

The internal movement of people across different provinces in South Africa and the net flows of in- and out-migration across the provinces have consequences on the prevalence and risk behaviors associated with non-communicable diseases [6]. These inter-provincial migrants are bound to face several circumstances while in transit and at destination. Issues of adaptation to ecological, economic, policy, political, and social circumstances are foremost, and could influence disease experiences [30]. Some of the migrants are exposed to health hazards along their journeys and might have difficulties accessing healthcare for policy reasons [7]. Studies have also shown that more migrants compared to non-migrants use primary healthcare [31] while [32] found that rural-urban migrants had decreased subjective well-being compared to the non-migrants between 2008 and 2012. Also, the study by [33] reported that both migrant and non-migrant African groups in South Africa were more vulnerable to depressive symptoms than Coloured and White migrants and non-migrants. Furthermore, [6] found that internal migrants generally had better mental health than the non-migrants. These risk factors as highlighted can cause these inter-provincial migrants to experience NCDs differently. Unfortunately, the issues of internal migration and NCDs have remained underexplored in literature, compared to the attention that is given to migrants’ and non-migrants’ experiences of infectious diseases like tuberculosis and HIV/AIDS [14].

Studies have also shown disparities in socioeconomic status, across provinces and districts, and most importantly, between urban and rural areas as well as between migrants and non-migrants in South Africa [34–36] Sex differences in CVDs resulting from sociocultural processes, such as differences in behaviors of women and men, different dietary habits, lifestyles or stress, and different attitudes toward treatments and prevention has been reported [37]. A recent study of inequalities among South Africans found that gender differences in NCDs was mainly accounted for by differences in characteristics rather than behavioral responses [38]. However, [38] observed that gender-based inequalities in NCDs are a stark reality in South Africa. It has been noted that sex differences in NCD risk factors need to be considered when evaluating one’s probability of developing NCD [39]. Earlier studies had reported that elderly people in South Africa have more NCD risk factors than younger people [40, 41] while other studies found that self-reported NCD multi morbidity was more common among women, at older ages, those having no or low levels of education, being separated, divorced or widowed, having higher household incomes, and among those from urban areas [42].

In a recent study, [43] posits that physical inactivity is a key risk factor of non-communicable diseases [44]. maintain that recreational football specifically decreases blood pressure and beneficial to NCDs related to cardiovascular and bone health, body composition, type 2 diabetes, and prostate cancer. According to [45], large family size and early-life farm exposure could be predisposing factors for asthma and rhinitis and respiratory symptoms among pre-school children in China. In addition, [46, 47] agree that smoking is a key risk factor for the development of asthma as avoiding tobacco smoke exposure during pregnancy might prevent or delay the development of asthma while [48] note that the prevalence of diabetes widely varied across provinces in Ecuador where higher rates were seen in provinces along the coastal region of the country.

Furthermore, [49] found higher prevalence of diabetes in coastal (8.2%) than in highlands (4.5%; *p* = 0.03), and jungle (3.5%; *p* < 0.02) regions of Peru. Thus, the need to take into account the contextual differences in studying the causes of increased NCD has been suggested [39]. In this context, non-communicable disease burden have been shown to vary between migrants and non-migrants, across different regions, city of residence and age groups [5, 41]. Kollamparambil and Booysen [38] stressed that ageing, race, urban residence, and region matters in NCDs prevalence. Most importantly, and central to this study is the fact that earlier studies have shown that inequalities in demographic and socioeconomic status results into significant marked geographical/spatial inequities in health outcomes such as NCDs especially between migrants and non-migrants [6, 38, 39, 50–53].

However, there remains a dearth of nationally representative analysis of the prevalence and contextual correlates of NCDs among inter-provincial migrants and non-migrants in South Africa. Therefore, the aim of the proposed study is to elucidate the prevalence and contextual correlates of NCDs among inter-provincial migrants and non-migrants in South Africa. The specific objectives among migrants and non-migrants are to; highlight the prevalence of non-communicable diseases (NCDs); and appraise the community, behavioral and individual risk factors of NCDs.

## Methods

### Data source and description of variables

Data was from the National Income Dynamics Study (NIDS), waves 5 (2017) which was the fifth wave of the longitudinal surveys of individuals and households living in the nine provinces of South Africa. For this study, a total of 28,055 respondents aged 15–64 years made up of 22,849 inter-provincial non-migrants and 5206 inter-provincial migrants were used. To generate the inter-provincial migration variable, all the respondents whose province of birth is the same with their province of residence were categorized as inter-provincial non-migrants and coded “0”. Conversely, the respondents whose province of birth was not the same with their province of residence were categorized as inter-provincial migrants and coded “1”. The study made use of a composite dependent/outcome of non-communicable disease, and two main categories of independent variables. The main categories of the independent variables were (i) community/ contextual-level variables, (ii) individual-level variables, and (iii) behavioral variables.

A composite index of NCD prevalence based on an earlier study, was generated from five variables of NCD in the dataset - diabetes, high blood pressure, stroke, asthma, and cancer based on responses from the respondents at the time of the survey. The questions asked in the NIDS dataset were “Have you ever been told by a doctor, nurse or health care professional that you have stroke, diabetes, high blood pressure, cancer, asthma? The responses to each of the five diseases were Yes/No. Based on the responses, these five variables of NCD was re-categorized as the outcome variable (NCD) which was used in the analysis. Respondents who reported having any or all of the five diseases were classified as having non-communicable disease while respondents who reported not having any of the five diseases were classified as not having non-communicable diseases [13]. The outcome variable for the study was therefore a binary variable of “Non-communicable disease (NCD)” and “No non-communicable disease (No NCD)”.

The community-level variables were place of residence (rural/urban residence), province of residence (the nine provinces of South Africa) and geographical location (coastal/ non-coastal provinces). The coastal provinces were Northern Cape, Eastern Cape, Western Cape and KwaZulu-Natal. On the other hand, the non-coastal provinces were Mpumalanga, Limpopo, Guateng, Free State and North West. The behavioral variables were smoking (yes/no) and engagement in physical exercise (yes/no). Finally the individual-level variables were gender (male/female), age (15–24 years/25–64 years/ 65+ years), race (Blacks/non-Blacks), income in Rands (< 5000/5000-10,000/> 10,000), education (primary/secondary/ tertiary), marital status (never married/ married/ widowed or divorced or separated), and household size (1–3 persons/4–6 persons/ 7+ persons).

### Data analysis

Before data analysis and based on earlier studies, the dataset was weighted for under sampling and oversampling errors [17]. In addition, all data analyses were based on inter-provincial migration status [17]. Univariate analysis was used to describe the characteristics of the study population, while bivariate analysis, which made use of Chi-Square, was used to interrogate for significant differences in the prevalence of non-communicable disease (NCD) between the contextual-level, behavioral-level and individual-level independent variables of the study. Furthermore, multilevel logistic regression models were used to estimate the influence of contextual-level, behavioral-level, and individual-level independent variables on occurrence of non-communicable diseases (NCD). The logistic regression, based on earlier literature was used since independent variable of “non-communicable disease (NCD)” was a binary categorical outcome of 1 and 0 [17].

For each of the inter-provincial migrant and inter-provincial non-migrant populations, there were five models. Model 1 was the empty/null model (no explanatory variable added) and according to an earlier study [17] had only a random intercept and was intended only to decompose the total variance into its individual, behavioral and contextual components and to identify the existence of possible contextual phenomenon for the non-communicable disease (NCD) outcome. In model 2, only the community-level explanatory variables were used to estimate the influence of contextual factors on the prevalence of non-communicable disease (NCD). Model 3 contained only individual-level explanatory variables and was used to estimate the influence of individual-level factors on the prevalence of non-communicable disease (NCD). Model 4 was used to estimate the influence of the behavioral-level factors on the prevalence of non-communicable disease (NCD). Finally, model 5 contained the contextual-level, behavioral-level, and individual-level factors and examined their combined effects on the prevalence of non-communicable disease (NCD). Furthermore, the fixed effects section of the models was made up of individual-level, behavioral-level, and contextual-level factors. All the regression analyses results were depicted as odds ratios (OR) at 95% confidence intervals (95% CI).

## Results

**Fig. 1:**
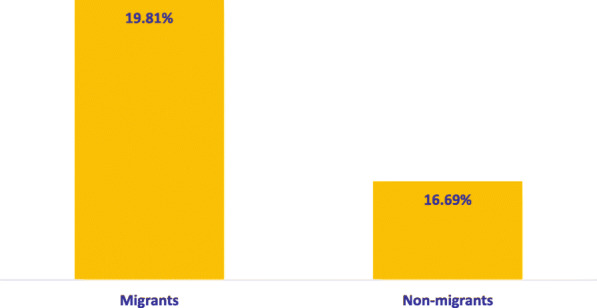
Prevalence of NCDs between migrants and non-migrants in the study area

The population of study had more females in the proportion of 58.45% for the migrants and 59.21% for the non-migrants. In addition, more of the sampled respondents were aged 25–64 years comprising of 73.15% of the migrant population and 61.52% of the non-migrant population (Table 1). While 51.23% of the migrant population had secondary education, compared to 58.61% of the non-migrant population secondary education, 54.48% of the migrants and 67.32% of the non-migrants earned less than R5, 000. Furthermore, 61.08% of the migrants and 75.27% of the non-migrants were never married while 70.03% of the migrants and 54.88% of the non-migrants were residents of urban areas. In addition, more migrants (19.81%) than non-migrants (16.69%) reported having NCD (Fig. 1).

**Table 1:** Population characteristics of the study area

Variables	Migrants	Non-Migrants
	***n(%)***	***n(%)***
**Gender**		
Male	2163 (41.55)	7391 (40.79)
Female	3043 (58.45)	10,727 (59.21)
**Age**		
15–24 years	746 (14.33)	5113 (28.22)
25–64 years	3808 (73.15)	11,146 (61.52)
65 + years	652 (12.52)	1859 (10.26)
**Race**		
Blacks	4218 (81.10)	14,440 (79.71)
Non-Blacks	983 (18.90)	3676 (20.29)
**Income (Rands)**		
< 5000	1065 (54.48)	3028 (67.32)
5000–10000	439 (22.46)	836 (18.59)
> 10000	451 (23.07)	634 (14.10)
**Education**		
Primary	1067 (20.56)	4533 (25.09)
Secondary	2659 (51.23)	10,588 (58.61)
Tertiary	1464 (28.21)	2945 (16.30)
**Marital Status**		
Never married	2773 (61.08)	12,115 (75.27)
Married	1493 (32.89)	3410 (21.19)
Div/wid/seperated	274 (6.04)	570 (3.54)
**Household Size**		
1–3 persons	2602 (49.98)	5907 (30.56)
4–6 persons	1797 (34.52)	7513 (38.87)
7+ persons	807 (15.50)	5909 (30.57)
**Exercise**		
No	3467 (66.60)	12,407 (68.48)
Yes	1739 (33.40)	5710 (31.52)
**Smoking**		
No	4279 (82.19)	14,900 (82.24)
Yes	927 (17.81)	3217 (17.76)
**Coastal Region**		
No	2814 (54.05)	8981 (39.31)
Yes	2392 (45.95)	13,868 (60.69)
**Residence**		
Rural	1560 (29.97)	10,298 (45.12)
Urban	3646 (70.03)	12,525 (54.88)
**Province**		
Western Cape	626 (12.02)	2960 (12.95)
Eastern Cape	433 (8.32)	2548 (11.15)
Northern Cape	248 (4.76)	1817 (7.95)
Free State	352 (6.76)	1158 (5.07)
KwaZulu-Natal	1085 (20.84)	6543 (28.64)
North West	300 (5.76)	1412 (6.18)
Gauteng	1286 (24.70)	3039 (13.30)
Mpumalanga	503 (9.66)	1542 (6.75)
Limpopo	373 (7.16)	1830 (8.01)

The bivariate results of prevalence of NCD among migrants in Table 2 showed significant differences in all the community, behavioral, and individual variables with the exception of household size. On the other hand, all the community, behavioral, and individual variables showed significant differences in prevalence of NCD among the non-migrants with the exception of respondents who smoke.

**Table 2:** Bivariate results of non-communicable diseases

Variables	Migrants		Non-Migrants	
	No (%)	Chi-square	No (%)	Chi-square
**Gender**		53.95***		235.9***
Male	292 (14.82)		788 (11.43)	
Female	621 (23.54)		1954 (20.48)	
**Age**		648.8***		2.231***
15–24 years	24 (3.30)		163 (3.26)	
25–64 years	586 (17.36)		1800 (17.94)	
65 + years	303 (59.88)		779 (55.80)	
**Race**		169.4***		265.3***
Blacks	600 (16.10)		1880 (14.31)	
Non-Blacks	312 (35.58)		862 (26.14)	
**Income (Rands)**		12.95**		20.81***
< 5000	116 (12.11)		403 (14.55)	
5000–10000	53 (13.15)		125 (16.71)	
> 10000	80 (19.37)		127 (22.16)	
**Education**		173.4***		849.8***
Primary	302 (35.66)		1203 (31.85)	
Secondary	353 (14.74)		1095 (11.07)	
Tertiary	255 (18.86)		438 (16.13)	
**Marital Status**		276.1***		15.87***
Never married	253 (9.81)		1030 (8.98)	
Married	382 (30.29)		926 (32.31)	
Div/wid/seperated	67 (30.32)		158 (33.05)	
**Household Size**		4.684		70.98***
1–3 persons	446 (18.90)		979 (19.94)	
4–6 persons	337 (21.59)		1054 (16.61)	
7+ persons	130 (18.92)		709 (13.69)	
**Exercise**		17.12***		106.4***
No	647 (21.60)		2080 (18.77)	
Yes	266 (16.50)		662 (12.37)	
**Smoking**		4.978*		0.815
No	768 (20.44)		2266 (16.81)	
Yes	145 (17.06)		476 (16.12)	
**Coastal Region**		8.512**		34.35***
No	460 (18.25)		902 (14.50)	
Yes	453 (21.70)		1840 (18.02)	
**Residence**		4.209*		27.06***
Rural	248 (17.97)		1276 (15.20)	
Urban	665 (20.60)		1466 (18.23)	
**Province**		64.24***		167.3***
Western Cape	148 (27.06)		412 (23.97)	
Eastern Cape	88 (23.16)		367 (18.64)	
Northern Cape	61 (29.19)		297 (21.79)	
Free State	79 (25.82)		160 (18.82)	
KwaZulu-Natal	156 (16.39)		764 (14.80	
North West	52 (19.55)		189 (18.31)	
Gauteng	179 (15.52)		243 (14.23)	
Mpumalanga	97 (21.37)		134 (11.78)	
Limpopo	53 (15.54)		176 (11.80)	

*p<0.05; **p<0.002; ***p<0.001

Table 3 shows the baseline/null models of NCD for the migrants and non-migrants without any explanatory variables. The intercepts of the null models revealed lower odds of NCD for both the migrants (0R = 0.24, 95%CI =0.22–0.28) and the non-migrants (0R = 0.20, 95%CI =0.18–0.22). In addition, the intracluster correlation coefficient (*ICC*) or *ρ* (the Greek rho) obtained were for non-migrants (6%) and for migrants (5%).

**Table 3:** The null model of non-communicable diseases

	***Migrants*** (Odds, 95% C.I)	Non-m***igrants*** (Odds, 95% C.I)
**_cons**	0.24 (0.22–0.28)*	0.20 (0.18–0.22)*
**Insig2u**	−1.80(−2.41 – −1.18)	−2.17(−2.66 - -1.68)
**Sigma_u**	0.41 (0.30–0.55)	0.34 (0.27–0.43)
**Rho**	0.05 (0.03–0.09)	0.06 (0.04–0.08)

Source: Authors’ analysis

*p<0.001

The introduction of community/contextual-level variables to NCD in models 2 in Table 4 did not change the reduced odds of NCD among the migrants (OR = 0.17, 95% CI-0.11-0.25) and non-migrants (OR = 0.13, 95%CI: 0.11–0.16). However, residents of coastal provinces showed higher odds of NCD for the migrants (OR = 1.99, 95%CI: 1.22–3.25) and the non-migrants (OR = 2.36, 95%CI: 1.76–3.17). In addition, the urban dwellers had higher odds of NCD than their rural counterparts with regard to the migrants OR = 1.10, 95%CI: 0.90–1.35) and the non-migrants (OR = 1.02, 95%CI: 0.91–1.15).

**Table 4:** Multilevel logistic analysis of predictors of non-communicable diseases

Variables	Model 2A contextual Migrants	Model 3A Individual migrants	Model 4A Behavioural Migrants	Model 5A Full Model Migrants	Model 2B contextual Non-migrants	Model 3B Individual Non-migrants	Model 4B Behavioural Non-Migrants	Model 5B Full model Non-migrants
***Individual***								
**Gender**								
Male		**1.00**		**1.00**		**1.00**		**1.00**
Female		1.42 (1.05–1.94)*		1.29 (0.92–1.79)		1.91 (1.57–2.32)*		1.82 (1.48–2.25)*
**Age**								
15–24 years		**1.00**		**1.00**		**1.00**		**1.00**
25–64 years		1.55 (0.65–3.70)		1.55 (0.65–3.70)		1.99 (1.24–3.18)*		2.02 (1.26–3.23)*
65 + years		5.80 (1.83–18.4)*		6.02 (1.88–19.3)*		11.3 (4.48–28.4(*		11.3 (4.46–28.3)*
**Race**								
Blacks		**1.00**		**1.00**		**1.00**		**1.00**
Non-Blacks		1.99 (1.40–2.82)*		1.99 (1.33–2.99)*		1.79 (1.41–2.26)*		1.57 (1.18–2.08)*
**Income (Rands)**								
< 5000		**1.00**		**1.00**		**1.00**		**1.00**
5000–10000		1.24 (0.83–1.85)		1.20 (0.79–1.82)		1.26 (0.96–1.64)		1.28 (0.98–1.67)
> 10000		1.42 (0.91–2.22)		1.39 (0.88–2.20)		1.63 (1.19–2.23)*		1.71 (1.25–2.34)*
**Education**								
Primary		**1.00**		**1.00**		**1.00**		**1.00**
Secondary		0.56 (0.35–0.89)*		0.51 (0.32–0.83)*		0.59 (0.45–0.77)*		0.58 (0.44–0.76)*
Tertiary		0.55 (0.33–0.92)*		0.49 (0.29–0.83)*		0.55 (0.40–0.76)*		0.54 (0.39–0.75)*
**Marital Status**								
Never married		**1.00**		**1.00**		**1.00**		**1.00**
Married		1.51 (1.06–2.15)*		1.46 (1.01–2.11)*		2.12 (1.71–2.64)*		2.08 (1.67–2.59)*
Div/wid/seperated		1.62 (0.90–2.89)		1.52 (0.84–2.74)		2.18 (1.50–3.17)*		2.20 (1.51–3.22)*
**Household Size**								
1–3 persons		**1.00**		**1.00**		**1.00**		**1.00**
4–6 persons		1.42 (1.02–1.97)*		1.41 (1.00–1.97)*		1.06 (0.86–1.32)		1.07 (0.86–1.33)
7+ persons		1.34 (0.78–2.30)		1.45 (0.83–2.52)		1.05 (0.81–1.36)		1.06 (0.82–1.39)
***Behavioral***								
**Exercise**								
No			**1.00**	**1.00**			**1.00**	**1.00**
Yes			0.72 (0.61–0.85)*	0.99 (0.71–1.37)			0.59 (0.54–0.66)*	1.07 (0.87–1.32)
**Smoking**								
No			**1.00**	**1.00**			**1.00**	**1.00**
Yes			0.76 (0.62–0.93)*	0.63 (0.41–0.97)*			0.80 (0.71–0.90)*	0.73 (0.57–0.94)*
***Community***								
**Coastal Region**								
No	**1.00**	**1.00**		**1.00**	**1.00**			**1.00**
Yes	1.99 (1.22–3.25)*			1.09 (0.48–2.49)	2.36 (1.76–3.17)*			2.53 (1.32–4.85)*
**Residence**								
Rural	**1.00**	**1.00**		**1.00**	**1.00**			**1.00**
Urban	1.10 (0.90–1.35)			1.83 (1.14–2.92)*	1.02 (0.91–1.15)			0.90 (0.70–1.16)
**Province**								
Western Cape	**1.00**	**1.00**		**1.00**	**1.00**			**1.00**
Eastern Cape	0.74 (0.48–1.15)			1.47 (0.81–2.69)	0.75 (0.58–0.96)*			0.76 (0.47–1.23)
Northern Cape	1.09 (0.68–1.77)			1.33 (0.62–2.84)	0.87 (0.67–1.14)			0.99 (0.63–1.55)
Free State	1.79 (1.06–3.02)*			**1.26 (0.49–3.24)**	1.72 (1.23–2.39)*			1.78 (0.87–3.66)
KwaZulu-Natal	0.54 (0.36–0.79)*			1.02 (0.64–1.90)	0.54 (0.43–0.68)*			0.57 (0.36–0.90)*
North West	1.31 (0.76–2.27)			1.47 (0.56–3.89)	1.65 (1.19–2.28)*			1.72 (0.84–3.49)
Gauteng	0.89 (0.54–1.45)			0.87 (0.39–1.92)	1.15 (0.84–1.59)			1.35 (0.70–2.62)
Mpumalanga	1.46 (0.86–2.47)			0.89 (0.36–2.16)	0.95 (0.67–1.36)			1.37 (0.66–2.85)
Limpopo	0.72 (0.60–0.95)			0.43 (0.28–0.61)	0.65 (0.41–0.98)			0.89 (0.74–1.05)
**_cons**	0.17 (0.11–0.25)*	0.07 (0.03–0.17)*	0.29 (0.25–0.33)*	0.05 (0.02–0.17)*	0.13 (0.11–0.16)*	0.05 (0.03–0.91)*	0.24 (0.22–0.27)*	0.04 (0.02–0.08)*
**Insig2u**	**−2.75(−3.63–1.87)**	**−13.3(−80–53.8)**	**−1.73(−2.34–1.12)**	**−13.7(−63.7–36.3)**	**−3.53(−4.24–2.24)**	**−2.40(−3.30–1.50)**	**−2.00(−2.48–1.53)**	**−2.72(−3.76–1.69)**
**Sigma_u**	**0.25 (0.16–0.39)**	**0.02 (0.00–0.05)**	**0.42 (0.31–0.57)**	**0.01 (0.00–0.04)**	**0.17 (0.12–0.24)**	**0.30 (0.19–0.47)**	**0.37 (0.29–0.47)**	**0.26 (0.15–0.43)**
**Rho**	**0.02 (0.01–0.04)**	**0.04 (0.02–0.07)**	**0.05 (0.03–0.09)**	**0.03 (0.01–0.05)**	**0.01 (0.00–0.02)**	**0.03 (0.01–0.06)**	**−.04 (0.02–0.06)**	**0.02 (0.01–0.05)**

^*^significant at 0.05 level of confidence

The inclusion of only individual-level factors of NCD in Models 3 still showed decreased odds of NCD for the migrants (OR = 0.07, 95%CI: 0–03-0.17) and the non-migrants (OR = 0.05, 95%CI: 0.03–0.91) in the study area. For both the non-migrant and migrant populations, marital status, increase in ages, the non-Blacks and the females were significantly associated with increased odds of NCD. Other factors that increased odds of NCD among migrants and non-migrants were larger household sizes and increased incomes. In Models 4, the behavioral factors of smoking and engaging in regular exercises were significantly associated with reduced odds of NCD among the migrants and the non-migrants. In the final model (Models 5) which included the individual-level, behavioral and community-level factors, the odds of NCD for the migrants (OR = 0.05, 95%CI-0.02-0.17) and the non-migrants (OR = 0.04, 95%CI-0.02-0.08) still remained on the decrease. In the migrant population, being aged 65+ years, the non-Blacks, being married households with 4–6 persons, and urban residents were significantly associated with increased odds of NCD. On the other hand, the variables with significantly increased odds for NCD among the non-migrants were the females, those aged 25+ years, the non-Blacks, people earning more than R10,000, the married, the widowed/divorced/separated, and the residents of coastal provinces.

## Discussion

From the results, more migrants reported NCDs compared to the non-migrants. This finding is supported by earlier studies which found health inequities between migrants and non-migrants [7, 9, 10, 15]. The differences in migrants and non-migrant health experiences have been attributed to prevailing social, cultural, political, and economic conditions, which migrants encounter during transit and settling down at their various destinations. Furthermore, [6] also noted that the internal migratory activities across different provinces in South Africa result in differences in the prevalence and risk behaviors associated with non-communicable diseases between migrants and non-migrants in the country.

Over the years, individual factors have been shown to influence the prevalence of NCDs among different populations [18–20] and this assertion is in tandem with the results of this study. In this context, this study found that among the migrants, the non-migrants, the females had more odds of NCDs compared to males, and this result is supported by literature [26]. This may be due to the fact that in most developing countries, women, especially the married ones, are known to live a relatively more sedentary life compared to the men. As such, they may not have enough exercise, which could affect their tendency to suffer more NCDs compared to the men. In addition, since most married women cater for then domestic affairs of their families, they usually worry more than the men with regard to the day-to-day-running of their homes, and this increases their stress, depression and anxiety levels. Furthermore, this study revealed that older people in the migrant and non-migrant populations had higher odds of NCDs relative to the younger people. This is corroborated by other studies, which noted that as people grow older, they are more predisposed to diseases and less disposed to rebuilding of their body cells which will strengthen their immune system against various diseases. In addition, most NCDs are usually associated with ageing populations than with younger populations [24–26]. Literature also show that better educated people had lower odds of NCDs as they are more likely to be knowledgeable on how to prevent and manage these diseases compared to their less educated counterparts [6, 26]. In agreement with these earlier studies, this study also found that both migrant and non-migrant populations with secondary and tertiary education had lower odds of NCDs compared to people with primary education. Finally, [29] found that larger family sizes have been responsible for the prevalence of NCDs in different populations. In agreement with this earlier study, this research reported that as the number of people in families increase among the migrants and non-migrants, the odds of NCDs also increased. This could be related to increased room-to-person density, which comes with unfavorable environmental conditions such as overcrowding, pollution, inadequate dietary and nutritional intakes because of large household sizes and inadequacy of food and nutrition etc., all of which could easily predispose such populations to NCDs.

This study also found significant differences in the influence of behavioral and community factors of the prevalence of NCDs among the migrants and the non-migrants in our study area. For instance, literature have shown that over the years, physical exercise have been associated with decreased odds of NCDs [27, 28]. According to these earlier studies, physical activities decreases blood pressure and is beneficial to NCDs related to cardiovascular and bone health, body composition, type 2 diabetes, and prostate cancer. In agreement to these earlier findings, this study found that both migrant and non-migrant population who were engaged in physical exercises reported lower odds of NCDs in the study area. However, smoking was associated with reduced odds of NCDs and this contradicts with earlier literature [15, 46, 47]. This inverse relationship between smoking and NCDs may be due to the fact that many of the country’s population start smoking from young ages and the predominantly cold weather of the country makes them to use smoking as a means of warming up their body due to the cold weather and not necessary because they usually deeply inhale the smoke. In addition, it may be due to the fact that majority of the sampled population smoke and it will may be difficult to attribute NCDs to everyone, as most of the sampled respondents who are smokers may not have gone for test and have been associated with NCDs. Finally, the results of this study showed that urban residents and people living in coastal provinces had higher odds of NCDs while significant differences were found in the odds of NCD across the different provinces among the migrant and non-migrant populations. The results of our study is corroborated by earlier literature which noted that while urban residents had higher odds of NCDs [22] diabetes incidence was higher in coastal areas [33]. This may be because most of the old people’s residences/homes for the elderly and retired persons, whose children cannot live with them, are usually situated along the coastal regions of the country. Moreover, the coastal regions are known for their cooling effects, which usually attract people suffering from different forms of illness to build homes and reside in such places, in order to benefit from the refreshing weather found in such locations. In addition, earlier studies showed that NCDs prevalence varied across different geographical/spatial regions [5, 23, 25, 34–37]. The reason for the health inequities between regions and places of residence, according to these earlier studies is attributable to inequalities in demographic, environmental and socioeconomic variables, which also affect the health status of these populations across the geographical and spatial areas.

## Conclusion

The results showed that differences exist in the prevalence of NCDs between migrants and non-migrants in the study area. With the exception of household size for migrants and smoking for non-migrants, the prevalence of NCDs showed significant differences in all the community, behavioral, and individual variables. Furthermore, the study found significant differences in the influence of individual, behavioral and community factors in the prevalence of NCDs among the migrant and non-migrant populations. The factors in the full model, which significantly increased odds of NCDs among the migrants and the non-migrants, were older populations, the non-Blacks, and higher education. Other factors, which significantly increased odds of NCDs among only the migrant population, were being married, household with 4–6 persons, and residents of urban areas. Conversely, living in coastal provinces, being a female, and people who earn more than 10,000 Rands were significantly associated with increased odds of NCDs among the non-migrant population.

These findings, therefore, emphasize the need for increased awareness campaigns, especially among the older populations on the preventive and mitigative strategies for NCDs. In addition, education of the population should be prioritized, as better-educated people were associated with decreased odds of NCDs among migrants and non-migrants. In addition, other means of reducing the prevalence of NCDs such as creation of more employment and income generating opportunities should be encouraged. Also, changes in lifestyles with regard to smoking and physical exercises should be more emphasized in specific contextual situations for the migrant and non-migrant populations, as highlighted by the results of this study.

### Limitation to the study

The dataset lacked the variable of alcohol intake, which could have been added to the behavioral factors of NCDs in the analysis. However, the variables of smoking and physical exercise were included in the study.

## Data Availability

The datasets used and analyzed during the current study are available at the National Income Dynamics Study (NIDS) repository, https://www.datafirst.uct.ac.za/dataportal/index.php/catalog/712

## Abbreviations

NIDS: National Income Dynamics Study
NCDs: Non-Communicable Diseases
LMICs: Low- and Middle-Income Countries
IOM: International Organization for Migration
CVDs: Cardiovascular Disease
NIDS: National Income Dynamics Study
*ICC*: Intracluster Correlation Coefficient

## References

[1] WHO (2019). Prevention and control of noncommunicable diseases in refugees and migrants: Technical guidance.

[2] Murray CJ, Vos T, Lozano R, Naghavi M, Flaxman AD, Michaud C (2012). Disability-adjusted life years (DALYs) for 291 diseases and injuries in 21 regions, 1990–2010: a systematic analysis for the global burden of disease study 2010. Lancet..

[3] Wagner KH, Brath HA (2012). Global view on the development of non-communicable diseases. Prev Med.

[4] WHO (2011). Diabetes Fact sheet N8312.

[5] Gouda HN, Charlson F, Sorsdahl K, Ahmadzada S, Ferrari AJ, Erskine H, et al. Burden of non-communicable diseases in sub-Saharan Africa, 1990–2017: results from the global burden of disease study 2017. Lancet Glob Health. 2019;7(10):e1375–87. 10.1016/S2214-109X(19)30374-2. 10.1016/S2214-109X(19)30374-2 31537368

[6] Ajaero CK, Odimegwu CO, Chisumpa V, Obisie-Nmehielle N. The influence of internal migration on mental health status in South Africa. Int J Ment Health Promot. 2017. 10.1080/14623730.2017.1327879.

[7] Javaweera H (2013). Migration and non-communicable diseases.

[8] International Organization for Migration (IOM). World Migration Report 2020. Geneva: IOM; 2019.

[9] Non communicable Disease Alliance (2009). Time to act: the global emergency of noncommunicable diseases. Report on “health and development: held back by noncommunicable diseases”. Meeting of the International Diabetes Federation, World Heart Federation and International Union Against Cancer during the 62nd World Health Assembly.

[10] WHO (2014). Global status report on noncommunicable diseases 2014.

[11] Ajaero CK, De Wet N, Odimegwu C. Integrating rural–urban differentials in the appraisal of prevalence and risk factors of non-communicable diseases in South Africa. GeoJournal. 2020. 10.1007/s10708-020-10282-5.

[12] Maimela E, Alberts M, Modjadji SEP, Choma SSR, Dikotope SA, Ntuli TS (2016). The prevalence and determinants of chronic non-communicable disease risk factors amongst adults in the Dikgale health demographic and surveillance system (HDSS) site, Limpopo Province of South Africa. PLoS One.

[13] Puoane T, Tsolekile L, Sanders D, Parker W (2008). Chronic non-communicable diseases: primary health care: programme areas. S Afr Health Rev.

[14] Oyebode O, Pape UJ, Laverty AA, Lee JT, Bhan N, Millett C (2015). Rural, urban and migrant differences in non-communicable disease risk factors in middle income countries: a cross sectional study of WHO-SAGE data. PLoS One.

[15] Ajaero CK, Odimegwu CO, Mkwananzi S, Banjo O (2018). Inter-provincial migration effect on youth risk behaviors in South Africa: A multilevel analysis of contextual- and individual-level factors. Cogent Soc Sci.

[16] Peer N (2015). The converging burdens of infectious and non-communicable diseases in rural-to-urban migrant sub-Saharan African populations: a focus on HIV/AIDS, tuberculosis and cardio-metabolic diseases tropical diseases. Travel Med Vaccines.

[17] Chen J (2011). Internal migration and health: re-examining the healthy migrant phenomenon in China. Soc Sci Med.

[18] Cui X, Rockett I, Yang T, Cao R (2012). Work stress, life stress, and smoking among rural-urban migrant workers in China. BMC Public Health.

[19] He X, Wong DFK (2013). A comparison of female migrant workers’ mental health in four cities in China. Int J Soc Psychiatry.

[20] Kuhn R, Barham T, Razzaque A, Turner P (2020). Health and well-being of male international migrants and non-migrants in Bangladesh: A cross-sectional follow-up study. PLoS Med.

[21] Donato KM, Caron L, Hamilton E (2020). Migration and mental health in Mexico: domestic migrants, return U.S. Migrants, and Non-Migrants. Front Psychiatry.

[22] Li X, Stanton B, Fang X, Lin D (2006). Social stigma and mental health among rural-to-urban migrants in China: a conceptual framework and future research needs. World Health Popul.

[23] Yang X, Derlega V, Luo H (2007). Migration, behaviour change and HIV/STD risks in China. AIDS Care.

[24] Yang X, Xia G (2008). Temporary migration and STD/HIV risky sexual behavior: a population-based analysis of gender differences in China. Soc Probl.

[25] Lin D, Li X, Wang B, Hong Y, Fang X, Qin X, et al. Discrimination, perceived social inequity, and mental health among rural-to-urban migrants in China. Community Ment Health J. 2011;47(2):171–80. 10.1007/s10597-009-9278-4. 10.1007/s10597-009-9278-4 PMC2891847 20033772

[26] Sudhinaraset M, Mmari K, Go V, Blum R (2012). Sexual attitudes, behaviours and acculturation among young migrants in Shanghai. Cult Health Sex.

[27] Gkiouleka A, Avrami L, Kostaki A, Huijts T, Eikemo TA, Stathopoulou T (2018). Depressive symptoms among migrants and non-migrants in Europe: Documenting and explaining inequalities in times of socio-economic instability. Eur J Pub Health.

[28] Gatt JM, Alexander R, Emond A, Foster K, Hadfield K, Mason-Jones A, et al. Trauma, resilience, and mental health in migrant and non-migrant youth: an international cross-sectional study across six countries. Psychiatry. 2020;10:997. 10.3389/fpsyt.2019.00997. 10.3389/fpsyt.2019.00997 PMC7073329 32210844

[29] Tzogiou C, Boes S, Brunner B (2021). What explains the inequalities in health care utilization between immigrants and non-migrants in Switzerland?. BMC Public Health.

[30] WHO (2018). Global health estimates 2016: Deaths by cause, age, sex, by country and by region, 2000–2016. Secondary Global health estimates 2016: Deaths by cause, age, sex, by country and by region, 2000–2016.

[31] Vearey J, de Gruchy T, Kamndaya M (2018). Exploring the migration profiles of primary healthcare users in South Africa. J Immigr Minor Health.

[32] Mulcahy K, Kollamparambil U (2016). The impact of rural-urban migration on subjective well-being in South Africa. J Dev Stud.

[33] Govera H, Bayat A. Differences in mental health among migrants and non-migrants in South Africa: evidence from the national income dynamics study in Dinbabo M F (Ed.). Afr Hum Mobility Rev. 2020;6(3):74–93.

[34] McIntyre D, Muirhead D, Gilson L (2002). (). Geographic patterns of deprivation in South Africa: informing health equity analyses and public resource allocation strategies. Health policy and. Planning..

[35] Noble M, Wright G (2013). Using indicators of multiple deprivation to demonstrate the spatial legacy of apartheid in South Africa. Soc Indic Res.

[36] Weimann A, Dai D, Oni T (2016). A cross-sectional and spatial analysis of the prevalence of multimorbidity and its association with socioeconomic disadvantage in South Africa: a comparison between 2008 and 2012. Soc Sci Med.

[37] Kautzky-Willer A, Harreiter J, Pacini G (2016). Sex and gender differences in risk, pathophysiology and complications of type 2 diabetes mellitus. Endocr Rev.

[38] Kollamparambil, U, Booysen F. Inequalities in non-communicable disease multi-morbidity among South Africans: a gender specific cross-sectional decomposition analysis. School of Economic and Business Sciences (SEBS), University of the Witwatersrand (Wits). Paper presented at the biennial conference of the Economic Society of South Africa (ESSA): 3–5 September, Johannesburg, South Africa. 2019; from https://www.datafirst.uct.ac.za/dataportal/index.php/citations/6743. Accessed 3 July 2020

[39] Boateng D, Agyemang C, Beune E, Meeks K, Smeeth L, Schulze M, et al. Migration and cardiovascular disease risk among Ghanaian populations in Europe: the RODAM study (research on obesity and diabetes among African migrants). Circ Cardiovasc Qual Outcomes. 2017;10(11):1–9. 10.1161/CIRCOUTCOMES.117.004013. 10.1161/CIRCOUTCOMES.117.004013 29150534

[40] Phaswana-Mafuya N, Peltzer K, Chirinda W, Musekiwa A, Kose Z (2013). Sociodemographic predictors of multiple non-communicable disease risk factors among older adults in South Africa. Glob Health Action.

[41] Rheeder P, Morris-Paxton AA, Ewing RG, Woods D (2017). The noncommunicable disease outcomes of primary healthcare screening in two rural sub districts of the eastern Cape Province, South Africa. Afr J Prim Health Care Fam Med.

[42] Camacho PA, Gomez-Arbelaez D, Otero J, GonzálezGómez S, Molina DI, Sanchez G, et al. Self-Reported prevalence of chronic non-communicable diseases in relation to socioeconomic and educational factors in Colombia: a community-based study in 11 Departments. Glob Heart. 2020. p. 15. 10.5334/gh.792. 10.5334/gh.792 PMC7218792 32489808

[43] Kramer A. An overview of the beneficial effects of exercise on health and performance. In: Xiao J, editor. Physical Exercise for Human Health. Advances in Experimental Medicine and Biology. Singapore: Springer; 2020. p. 1228. 10.1007/978-981-15-1792-1_1. 10.1007/978-981-15-1792-1_1 32342447

[44] Sarmento H, Clemente FM, Marques A, Milanovic Z, Harper LD, Figueiredo A (2019). Recreational football is medicine against non-communicable diseases: a systematic review. Scand J Med Sci Sports.

[45] Norback D, Lu C, Wang J, Zhang Y, Li B, Zhao Z, et al. Asthma and rhinitis among Chinese children- indoor and outdoor air pollution and indicators of socioeconomic status (SES). Environ Int. 2018;115:1–8. 10.1016/j.envint.2018.02.023. 10.1016/j.envint.2018.02.023 29529393

[46] Grabenhenrich LB, Gough H, Reich A, Eckers N, Zepp F, Nitsche O (2014). Early-life determinants of asthma from birth to age 20 years: a German birth cohort study. Allergy Clin Immunol.

[47] Furuhata M, Otsuka Y, Kaneita Y, Nakagome S, Jike M, Itani O, et al. Factors associated with the development of childhood asthma in Japan: a Nationwide longitudinal study. Matern Child Health J. 2020;24(7):911–22. 10.1007/s10995-020-02944-0. 10.1007/s10995-020-02944-0 32342275

[48] Orces CH, Lorenzo C (2018). Prevalence of predibetes and diabetes among older alduts in Ecuador: analysis of the SABE survey. Diabetes Metab Syndr Clin Res Rev.

[49] Seclen SN, Rosas ME, Arias AJ, Huayta E, Medina C (2015). Prevalence of diabetes and impaired fasting glucose in Peru: report from PERUDIAB, a national urban population based longitudinal study. BMJ Open Diabetes Res Care.

[50] Adebayo SB, Gayawan E, Ujuju C, Ankomah A (2013). Modelling geographical variations and determinants of use of modern family planning methods among women of reproductive age in Nigeria. J Biosoc Sci.

[51] Kazembe LN (2013). A Bayesian two part model applied to analyze risk factors of adult mortality with application to data from Namibia. PLoS One.

[52] Atari DO, Mkandawire P (2014). Spatial variation of management of childhood diarrhea in Malawi. Health Place.

[53] Gayawan E, Turra CM (2015). Mapping the determinants of child mortality in Nigeria: estimates from mortality index. Afr Geogr Rev.

